# Supplementary material for: Identification of an Individualized Prognostic Biomarker for Serous Ovarian Cancer: A Qualitative Model
Source: Diagnostics (Basel). 2022 Dec 12;12(12):3128. doi: 10.3390/diagnostics12123128 (PMC9777083; doi:10.3390/diagnostics12123128)
Supplement: Supplementary file 1 [file diagnostics-12-03128-s001.zip › diagnostics-2027900-supplementary.pdf]

Table S1. Tumor response to chemotherapy drugs in TCGA serous ovarian cancer samples.

| Response | High (All drug) | Low (All drug) | High (Platinum) | Low (Platinum) |
|---|---|---|---|---|
| CR[*] | 78 | 154 | 74 | 141 |
| PR | 29 | 17 | 26 | 16 |
| PD | 20 | 12 | 19 | 11 |
| SD | 16 | 12 | 14 | 9 |
| Unknown | 58 | 70 | 32 | 57 |

[*]CR, complete response; PR, partial reponse; SD, stable disease; PD, progressive disease.

Table S3. SOV-P20 and its correlation with serous ovarian cancer.

| Gene Symbol | Relation to serous ovarian cancer | PMID |
|---|---|---|
| FSTL3>THOP1 | - | |
| DHRS7>DLG3 | - | - |
| PDE4B>FOXJ1 | - | - |
| TEX261>PYCR1 | - | - |
| IGFBP6>PLAGL2 | IGFBP family impact on serous ovarian cancer; PLAGL2 axis mediated ARAP1-AS1 to promote tumorigenesis in ovarian cancer by promoting cell proliferation, migration, and invasion. | 33282953; 34149172 |
| NPC1>PIR | - | - |
| LY96>SLC1A5 | - | - |
| CPVL>SETD4 | - | - |
| PLXNC1>EPHA1 | - | - |
| ITGB8>THAP3 | ITGB8 expression increased with DNA amplification in patients with high-grade serous ovarian cancer, significantly reducing overall and progression-free survival. | 30531684 |
| ZHX3>NUP160 | - | - |
| TCF15>HPCA | - | - |
| AFAP1>LAMP3 | Epithelial ovarian cancer; high expression of LAMP3 was significantly associated with poor survival outcomes. | 29526252 |
| SOX1>LCE2B | SOX1 methylation in patients with ovarian cancer; the higher the risk of recurrence, the shorter the survival time. | 18942711 |
| CSF2>ADAM20 | - | - |
| ALOX12P2>ATP2C2 | - | - |
| TMEM47>SCRIB | SCRIB as a prognostic biomarker in ovarian cancer. | 32564009 |
| TEX261>FOXJ1 | - | - |
| CST6>SLC1A5 | SLC1A5 expression was associated with a worse prognosis in FIGO stage I–II epithelial ovarian cancer. | 28609484 |
| GPRC5B>CXCL11 | High-expression CXCL11 serous ovarian cancer had a worse prognosis. | 30858874 |

Table S4. Existing models for predicting serous ovarian cancer prognosis.

| **Study** | **Data source** | **Gene signature** | **PMID** |
|---|---|---|---|
| Buttarelli, 2020 | Clinical Trials | MEG3 | 32295169 |
| Zhang, 2020 | Clinical Trials | MAP3K3 | 32269720 |
| Liu, 2020. | GEO | 5-gene | 33281878 |
| Zheng, 2020 | TCGA | 11-gene | 33271935 |
| Liu, 2018 | GEO | 7-gene | 30585265 |
| Pan, 2020 | TCGA | 6-gene | 33193589 |
| Zhang, 2020 | TCGA | 8-gene | 32880385 |
| Liu, 2016 | TCGA | 5-gene | 27478834 |
| Zhang, 2018 | TCGA | 2-gene | 29456732 |
| Zhou, 2019 | TCGA | 3-gene | 31579405 |
| Wang, 2019 | TCGA; GEO | 6-gene | 31423184 |
| An, 2018 | TCGA; GEO | 8-gene | 30410611 |
| Wang, 2019 | TCGA; GEO | 5-gene | 30569721 |
| Yang, 2021 | TCGA; GEO | 7-gene | 34966691 |
| Wang, 2020 | TCGA; GEO | 8-gene | 32186777 |
| Zhang, 2021 | TCGA; GEO | 17-gene-pairs | 33543590 |
| Shen, 2019 | TCGA; GEO | 129-gene | 30594555 |
| Yang, 2016 | TCGA | 19-gene | 27059002 |
| Willis, 2016 | TCGA; HAS | 32-gene | 26886260 |
| Wang, 2017 | TCGA; GEO | 10-gene | 27846619 |
| Sabatier, 2011 | TCGA | 7-gene | 21654678 |
| Millstein, 2020 | GEO | 101-gene | 32473302 |
| Lin, 2021 | TCGA; GEO | 35-gene-pairs | 33680950 |
| Zhang, 2019 | TCGA; GEO | 20-gene-pairs | 31695415 |
